# LFP-guided targeting of a cortical barrel column for *in vivo* two-photon calcium imaging

**DOI:** 10.1038/srep15905

**Published:** 2015-10-29

**Authors:** Joon-Hyuk Lee, Hee-Sup Shin, Kwang-Hyung Lee, Sooyoung Chung

**Affiliations:** 1 Department of Bio and Brain Engineering, Korea Advanced Institute of Science and Technology (KAIST), Daejeon 305-701, Republic of Korea; 2 Center for Neuroscience, Korea Institute of Science and Technology (KIST), Seoul 136-791, Republic of Korea; 3 Center for Cognition and Sociality, Institute for Basic Science (IBS), Daejeon 305-811, Republic of Korea

## Abstract

Two-photon microscopy of bulk-loaded functional dyes is an outstanding physiological technique that enables simultaneous functional mapping of hundreds of brain cells *in vivo* at single-cell resolution. However, precise targeting of a specific cortical location is not easy due to its fine dimensionality. To enable precise targeting, intrinsic-signal optical imaging is often additionally performed. However, the intrinsic-signal optical imaging is not only time-consuming but also ineffective in ensuring precision. Here, we propose an alternative method for precise targeting based on local field potential (LFP) recording, a conventional electrophysiological method. The heart of this method lies in use of the same glass pipette to record LFPs and to eject calcium dye. After confirming the target area by LFP using a glass pipette, the calcium dye is ejected from the same pipette without a time delay or spatial adjustment. As a result, the calcium dye is loaded into the same ensemble of brain cells from which the LFP was obtained. As a validation of the proposed LFP-based method, we targeted and successfully loaded calcium dye into layer 2/3 of a mouse barrel column.

Two-photon fluorescence microscopy^1^,^2^ is one of the most advanced techniques used to visualize the structure and function of brain cells *in vivo*. Because its spatial resolution is less than 1 μm^3^, it allows individual brain cells or even subcellular structures such as dendritic spines to be imaged. In combination with bulk-loaded functional dyes, such as calcium indicators, it enables simultaneous functional mapping of hundreds of brain cells at single-cell resolution from various cortical depths *in vivo*^3^,^4^,^5^,^6^,^7^,^8^,^9^,^10^,^11^. Several review papers have discussed this attractive imaging technique in greater detail^12^,^13^,^14^,^15^.

Despite its virtues, *in vivo* two-photon microscopy suffers due to the fine dimensionality of the imaged area. It can reveal functional structures at single-cell resolution, but only at the expense of the spatial extent. A small structure that exists in low density over the cortex is difficult to target by two-photon microscopy. For example, a cortical column typically spans only a few hundred micrometres^16^,^17^,^18^. Without additional technical assistance, it is not easy to target such a small structure over the cortex.

Intrinsic-signal optical imaging is the method of choice to target a small cortical structure with two-photon microscopy^7^,^19^,^20^. The target structure can be identified as a dark spot on a single-condition map or on a contrast map using subtraction or division. The location of the target structure is identified on the cortical surface by the use of surface blood vessel patterns. The optical method is non-invasive. It reliably locates the target structure over a wide cortical area. However, it has a few problems. The optical map takes a long time to obtain. It does not have depth information, which makes it suitable mostly for columnar structures. The low spatial resolution (>100 μm) combined with optical blurring may induce a systematic positional error^21^. Optical imaging using voltage-sensitive dyes could overcome some of these drawbacks, but this method is hardly used *in vivo* due to the toxicity of voltage-sensitive dyes. Volumetric-signal optical imaging could produce a larger signal but performs similarly in other aspects^22^.

Localization of a target area, moreover, does not guarantee successful calcium imaging. Precise ejection of the calcium dye into the target area in also required. In the optical method, after target localization, the pipette containing the calcium dye is inserted into the cortex and advanced towards the target area. The pipette can miss the target if the displacement of the insertion point from the target location over the cortical surface is significantly miscalculated; this displacement can be estimated from a few factors including the angle of the pipette, the slope of the cortical surface, and the ideal cortical depth for dye ejection. When all of the potential errors and problems of the optical targeting method are combined, the pipette can miss the target by up to a few hundred micrometres (personal communications, K. Ohki and S. Chung).

Here, we propose an alternative method based on local field potential (LFP) recording. LFP recording is a widely used conventional electrophysiological method. LFPs are low-frequency (<300 Hz) extracellular electrical signals that reflect the activity of the ensemble of neurons around the electrode^23^,^24^. LFP recording is often used to provide information about current flow or to pinpoint the location of massive coherent neuronal activity, such as the laminar location of thalamic synaptic inputs within a cortical column^25^,^26^,^27^. The usefulness of LFPs in providing such information greatly depends on the extent of spatial summation. Recently, two independent studies on cat and monkey visual cortex concluded that LFPs originated more locally than previously thought and were summed from a volume of neuronal tissue with a radius of no more than 250 ~ 500 μm^28^,^29^. LFP recording, therefore, can potentially be used to monitor the activity from a small cortical structure^30^,^31^,^32^,^33^,^34^, such as an anatomical or functional cortical column, which typically span a few hundred micrometres at most^16^,^17^.

The proposed LFP-based targeting has the additional benefit of ensuring precise dye ejection to the localized target area. The target area is localized by LFP recording using a glass pipette filled with calcium indicator dye^33^. The calcium dye is then immediately ejected from the same pipette with no time delay; there is no need to withdraw the pipette from the cortex or insert a new pipette into the cortex. Therefore, the calcium dye can be ejected into the same population of neurons from which the LFP was obtained. This method can overcome most of the problems of intrinsic-signal optical imaging described earlier and provides faster and more precise targeting of a small cortical area for *in vivo* two-photon microscopy.

We used the LFP-based method to target a specific barrel column in the mouse somatosensory cortex. The results demonstrated the validity and usefulness of the LFP-based method to provide precise targeting for *in vivo* two-photon calcium imaging using bulk-loaded functional indicator dyes.

## Results

### Localization of the target barrel column by LFP

**Figure 1:**
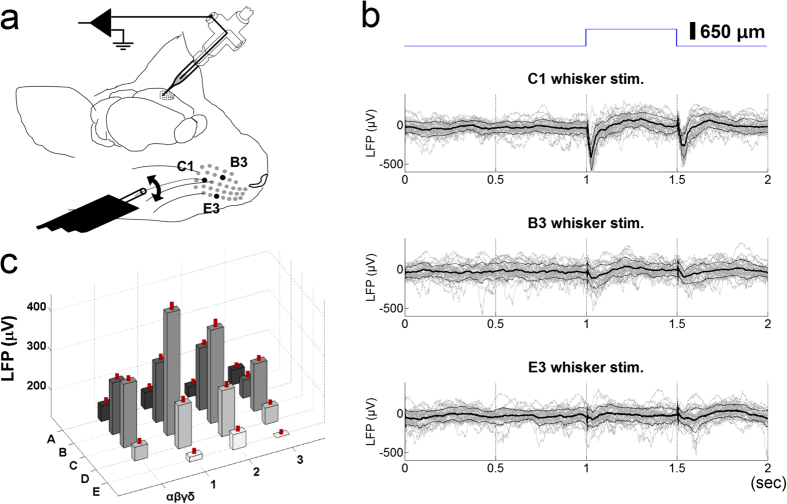
Localization of the C1 barrel by LFP. (**a**) LFP recording setup. The piezo wafer (shown as a black plate) deflected each of the whiskers while LFP signals were recorded from layer 2/3 of the barrel column. The picture was drawn by J-H. Lee. (**b**) Typical LFPs evoked by stimulation of the PPW (C1) and two non-PPWs (B3 & E3). The stimulated whiskers are indicated by filled dots in Fig. 1a. The blue trace on the top shows the whisker deflection. The lower 3 panels show the LFP responses; 30 individual traces are shown in grey; the averaged trace, in black; and the S.E.M., in dark grey. (**c**) Summary of the LFP responses evoked by all of the whisker stimulations. For clarity, the peak amplitude is presented as absolute value. The S.E.M. is indicated as a vertical red line on each bar.

The magnitude of the sensory-evoked LFP signals could indicate whether the pipette reached the target barrel column. A schematic view of the LFP recording is shown in Fig. 1a. The LFP signals of the targeted barrel column, the C1 barrel column, and the surrounding barrel columns are shown. The typical LFP response to a piezo-controlled whisker deflection is shown in Fig. 1b. Using a 650-μm deflection near the base of the whiskers, the C1 whisker evoked the strongest LFP response, while the neighbouring whiskers (B3 and E3) evoked weaker LFP responses. The LFP responses to all of the stimulated whiskers were summarized in Fig. 1c. Compared to the C1 whisker, all of the other whiskers evoked significantly smaller LFP responses (two-tailed Student’s t-test; β: p < 0.00001, C2: p = 0.00832, D1: p < 0.00001, D2: p < 0.00001; Mann-Whitney rank sum test for the other whiskers, p < 0.00001 for all pairs). The largest response was found in the centre, corresponding to the C1 whisker; the responses for the other whiskers gradually decreased as a function of the distance between the stimulated whisker and the C1 whisker. This general response pattern was observed in single unit recordings^35^ as well as in subthreshold intracellular responses^36^,^37^. This response pattern indicates that the LFP recording was likely being made in the C1 whisker barrel column^33^,^38^,^39^. A similar response pattern was also observed for the latency of peak LFP (see Supplementary Figure S1). Thus, we defined C1 as the presumed principal whisker (PPW) of the recording area and the other whiskers as non-presumed principal whiskers (non-PPWs).

In summary, the target C1 barrel column could be localized based on the LFP signal pattern evoked by the whisker stimulations.

### Calcium signals evoked by PPW and non-PPW deflection

**Figure 2:**
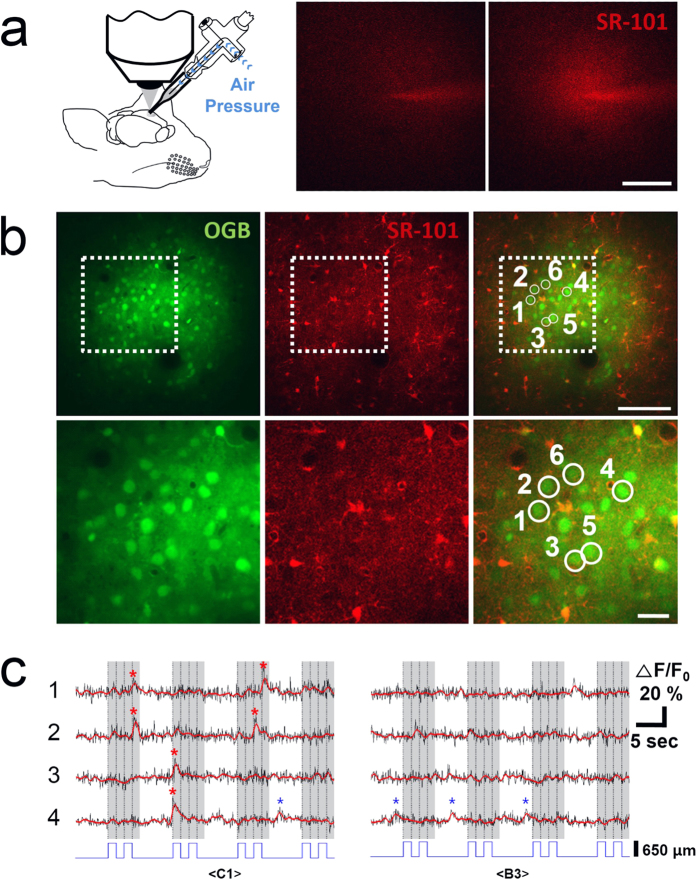
Dye loading and neuronal Ca^2+^ responses. (**a**) Dye ejection setup. After localizing the target area 270 μm below the pial surface by LFP, the calcium dye was ejected by air pressure without any displacement of the pipette. The right two panels show the spread of the dye before (middle panel) and during (right panel) ejection, as indicated by SR-101. Scale bar, 100 μm. The picture was drawn by J–H. Lee. (**b**) Images of OGB-1AM (left) and SR-101 (middle) as well as the superimposed image (right) at a depth of 180 μm. The lower row shows magnified images. Scale bar, 100 μm in the upper panel and 20 μm in the lower panel. (**c**) Neuronal Ca^2+^ responses to PPW (C1, left) and non-PPW (B3, right) stimulation. The numbers on the left indicate the neurons numbered in (**b**). Raw Ca^2+^ signals (black traces) were low-pass filtered (red traces superimposed; Butterworth; cut-off frequency of 0.8 Hz). The asterisks indicate the events where the Ca^2+^ responses exceeded the threshold, which was 1.5 times the standard deviation of the Ca^2+^ signals of all off periods. The events that occurred during whisker deflection (on period) are marked with red asterisks, and the spontaneous events that occurred between whisker deflections (off period) are marked with blue asterisks. The whisker stimulation protocol is shown in the blue trace at the bottom. Shaded and unshaded regions indicate the on and off periods of stimulation. The image frames (512 × 16) were obtained at 13.16 Hz.

Immediately after localizing the target barrel column by LFP recording, the calcium indicator dye was ejected through the same glass pipette without any spatial adjustment. A schematic view of dye ejection is shown in Fig. 2a (left). The spread of calcium dye was monitored by SR-101 as shown in Fig. 2a (middle: before ejection; right: during ejection). A gradual, symmetric circular spread of SR-101 is regarded as a good ejection.

The ejected calcium dye was loaded into a cortical volume with a diameter of 300 ~ 400 μm (Fig. 2b), which is comparable to the size of a mouse barrel column. Figure 2b shows the neurons and astrocytes loaded with OGB-1AM (left) and the astrocytes loaded with SR-101 (middle)^40^. The black circles that occasionally appear in the OGB-1 AM image are blood vessels. The superimposed image (right) clearly separates neurons (round cells in green) from astrocytes (irregularly shaped cells in orange). Only neurons were included in further analysis.

Ca^2+^ responses were evoked by stimulation of the PPW and non-PPWs. Figure 2c shows the raw Ca^2+^ signals evoked by PPW (C1 whisker, left) and non-PPW (B3 whisker, right) stimulation in 4 neurons marked from 1 to 4 in Fig. 2b. Ca^2+^ transients were detected as supra-threshold Ca^2+^ responses (see Methods) and marked with asterisks: red for sensory-evoked Ca^2+^ transients and blue for spontaneous Ca^2+^ transients. Six sensory-evoked and 1 spontaneous Ca^2+^ transients occurred during PPW stimulation. In contrast, no sensory-evoked and 3 spontaneous Ca^2+^ transients occurred during non-PPW stimulation. All of the spontaneous Ca^2+^ transients were smaller in magnitude than the PPW-evoked Ca^2+^ transients. Therefore, even though the responses were not reliable, PPW stimulation tended to evoke Ca^2+^ transients that were stronger and more frequent than those evoked by non-PPW stimulation.

**Figure 3:**
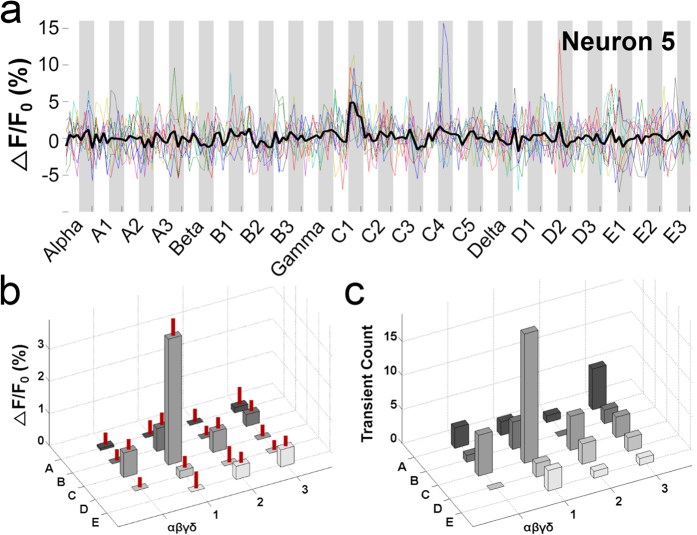
A neuron with sharp whisker tuning. (**a**) Averaged Ca^2+^ signals of Neuron 5 in Fig. 2b in response to stimulation of 21 whiskers. Ten individual traces are shown in colour; the average trace is depicted by the thick black line and is superimposed over the individual traces. The image frames (512 × 512) were obtained at 0.76 Hz. The stimulated whiskers are listed on the x-axis in an arbitrary order. The shaded and unshaded regions indicate the on and off periods of stimulation. (**b**) Topographical 3D plot of Ca^2+^ signals against stimulated whiskers. The Ca^2+^ signals evoked by C1 stimulation were significantly larger than those evoked by stimulation of the other whiskers (one-way ANOVA, F_(18,741)_ = 7.214, p < 0.001; Holm-Sidak method for multiple comparisons between the C1 response and the other responses, p < 0.001 for all pairs). The S.E.M. is indicated as a vertical red line on each bar. Negative Ca^2+^ signal values are represented as zero for clarity. (**c**) Topographical 3D plot of transient counts against stimulated whiskers. The transients were detected as Ca^2+^ signals that were larger than the threshold and that occurred during the on periods.

**Figure 4:**
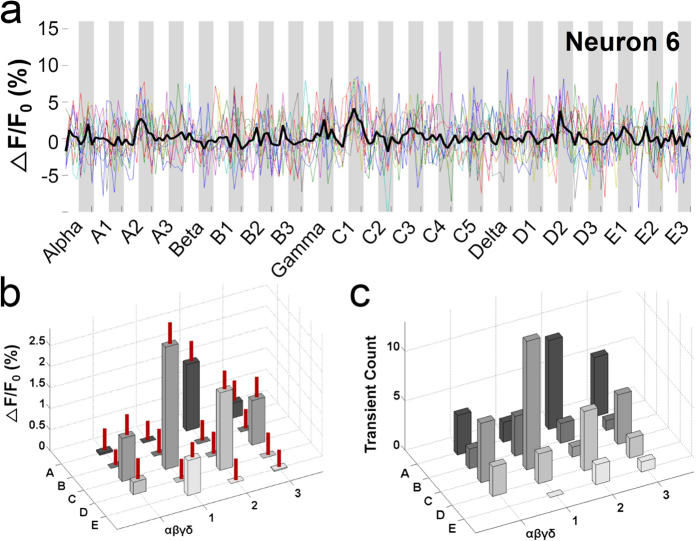
A neuron with broad whisker tuning. (**a**) Averaged Ca^2+^ signals of Neuron 6 in Fig. 2b in response to stimulation of 21 whiskers. (**b**) Topographical 3D plot of Ca^2+^ signals against stimulated whiskers. The S.E.M. is indicated as a vertical red line on each bar. This neuron was tuned not only to the C1 whisker but also to other whiskers, shown as multiple peaks in Ca^2+^ responses (one-way ANOVA, F_(18,741)_ = 4.342, p < 0.001; Holm-Sidak method for multiple comparisons between the C1 response and the other responses; α: p = 0.003; A1: p < 0.001; A2: p = 0.996; A3: p = 0.021; β: p < 0.001; B1: p < 0.001; B2: p = 0.001; B3: p < 0.001; γ: p = 0.501; C2: p < 0.001; C3: p = 0.554; δ: p = 0.015; D1: p = 0.002; D2: p = 1; D3: p < 0.001; E1: p = 0.244; E2: p < 0.001; and E3: p = 0.003). (**c**) Topographical 3D plot of transient counts against stimulated whiskers.

**Figure 5:**
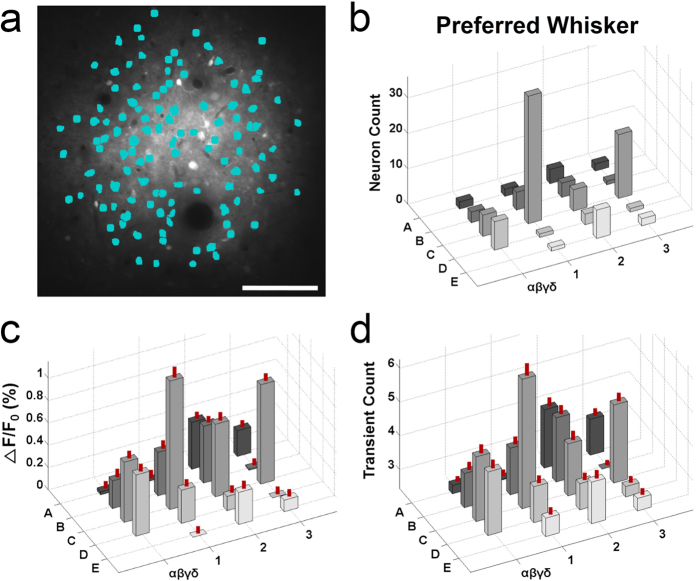
Population analysis of Ca^2+^ responses. (**a**) A total of 113 neurons were detected (cyan dots) from the image shown in Fig. 2b after astrocytes (white & grey dots) were excluded. Imaging depth, 180 μm; Scale bar, 100 μm. (**b**) Topographical 3D plot of the distribution of the preferred whisker of individual neurons. The largest group of neurons (36/113) preferred the C1 whisker. (**c**) Topographical 3D plot of the average Ca^2+^ signals from all of the detected neurons. The Ca^2+^ signals evoked by C1 whisker stimulation were significantly larger (one-way ANOVA, F_(20,2352)_ = 24.741, p < 0.001; Holm-Sidak method for multiple comparisons between C1 responses and the other responses, p < 0.001 for all pairs) than those evoked by stimulation of the other whiskers, except the C3 whisker (p = 0.311). The S.E.M. is indicated as a vertical red line on each bar. (**d**) Topographical 3D plot of the average transient counts from the total population of neurons. The Ca^2+^ transients observed in response to C1 whisker stimulation were significantly more frequent than those in response to stimulation of the other whiskers (one-way ANOVA, F_(20,2352)_ = 25.454, p < 0.001; Holm-Sidak method for multiple comparisons between the C1 response and the other responses, p < 0.001 for all pairs).The S.E.M. is indicated as a vertical red line on each bar.

To verify that the PPW was indeed the principal whisker, we examined the Ca^2+^ responses to PPW or non-PPW stimulation of both individual neurons (Figs 3 and 4) and the ensemble of neurons (Fig. 5).

First, we examined the Ca^2+^ responses of individual neurons to stimulation of the PPW and non-PPWs. Neurons from the same column showed differing degrees of whisker preference; some neurons exhibited a sharp preference, while others did not. Figure 3 shows an example neuron with relatively sharp whisker tuning (the neuron marked as 5 in Fig. 2b). Again, PPW stimulation evoked larger and more reliable calcium responses than non-PPW stimulation (Fig. 3a,b). Although the tuning was not clear in the individual traces (coloured traces) due to noise, it became obvious in the averaged trace (n = 10, thick black trace) and was statistically significant (one-way ANOVA, F_(18,741)_ = 7.214, p < 0.001; Holm-Sidak method for multiple comparisons between the C1 response and the other responses, p < 0.001 for all pairs). This sharp tuning to the PPW was also obvious in calcium transient counts (Fig. 3c). A neuron with broad whisker tuning is shown in Fig. 4 (the neuron marked as 6 in Fig. 2b). This neuron responded to stimulation of the PPW and a few non-PPWs, such as A2, C3, D2 and γ; this response pattern can be seen as multiple peaks in the averaged calcium responses (Fig. 4a, one-way ANOVA, F_(18,741)_ = 4.342, p < 0.001; Holm-Sidak method for multiple comparisons between the C1 response and the other responses; α: p = 0.003; A1: p < 0.001; A2: p = 0.996; A3: p = 0.021; β: p < 0.001; B1: p < 0.001; B2: p = 0.001; B3: p < 0.001; γ: p = 0.501; C2: p < 0.001; C3: p = 0.554; δ: p = 0.015; D1: p = 0.002; D2: p = 1; D3: p < 0.001; E1: p = 0.244; E2: p < 0.001; and E3: p = 0.003). This response pattern is evident in the topographical 3D plots of calcium response magnitude (Fig. 4b) and calcium transient counts (Fig. 4c).

The ensemble response to whisker stimulation was obtained from all 113 neurons detected (Fig. 5). The astrocytes (white & grey dots) were separated from the neurons (cyan dots) and excluded from the analysis (Fig. 5a). The preferred whisker, which evoked the strongest calcium signal, was determined for each neuron, and this distribution was plotted (Fig. 5b). The results indicated that the PPW was the most preferred (36 out of 113 neurons) (Fig. 5b). The Ca^2+^ signals and transient counts summed over all 113 neurons are shown in Fig. 5c,d. The ensemble of neurons responded not only to PPW stimulation but also to non-PPW stimulation. PPW stimulation, however, evoked significantly larger Ca^2+^ signals compared to non-PPW stimulation (Fig. 5c, one-way ANOVA, F_(20,2352)_ = 24.741, p < 0.001; Holm-Sidak method for multiple comparisons between the C1 response and the other responses, p < 0.001 for all pairs), excluding C3 whisker stimulation (p = 0.311). With respect to transient counts, PPW stimulation evoked significantly more calcium transients than all of the non-PPW stimulations (Fig. 5d, one-way ANOVA, F_(20,2352)_ = 25.454, p < 0.001; Holm-Sidak method for multiple comparisons between the C1 response and the other responses, p < 0.001 for all pairs). This result is consistent with previous studies that showed that even though neurons in the same barrel column exhibited different receptive fields^33^,^35^, the average Ca^2+^ response in the barrel column was strongest in response to principal whisker stimulation^20^. This indicates that the PPW (C1 whisker) was the true principal whisker of the dye-loaded area.

In summary, various measurements of the averaged Ca^2+^ responses recorded from all of the detected neurons in unison showed that the strongest response was obtained in response to PPW stimulation, indicating that the PPW was the true principal whisker of the dye-loaded area. Therefore, the proposed method was successfully applied to target a specific barrel column for *in vivo* two-photon imaging using bulk-loaded calcium dye.

### Strong correlation between the LFPs and calcium signals

**Figure 6:**
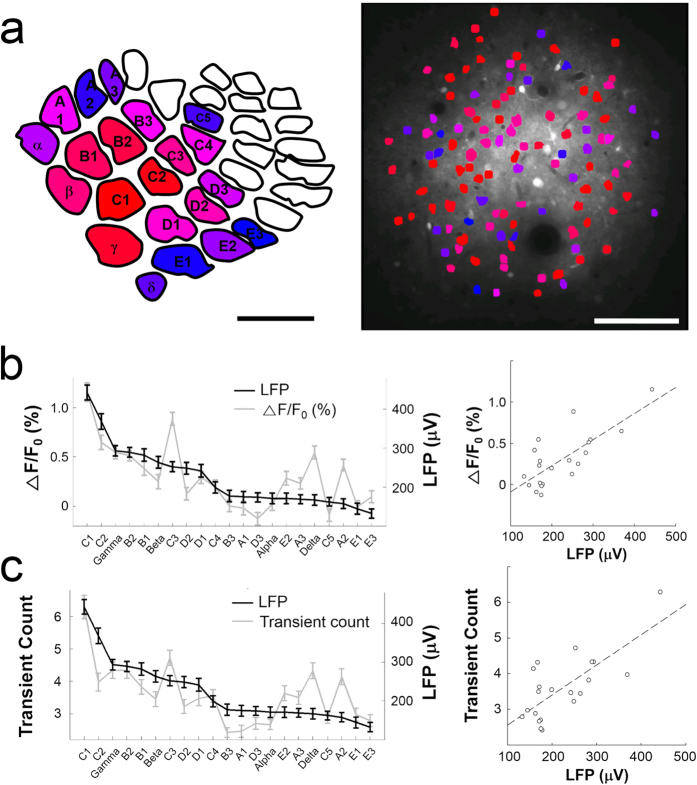
Correlation between LFPs and Ca^2+^ responses. (**a**) A normative topographical barrel map^41^ coloured in a red-blue scale according the magnitude of the LFPs each whisker evoked (left panel). Scale bar, 500 μm. All 113 detected neurons were coloured according to the colour map in the left panel based on their preferred whisker (right panel). The neurons that preferred the whisker that evoked largest LFP (C1) are coloured in red. The large number of neurons in reddish colours indicates a tendency towards a preference for the whiskers that evoked relatively large LFPs. Individual neurons tended to show larger Ca^2+^ signals to the whiskers that evoked larger LFPs. Scale bar, 100 μm. (**b**) Plot of the Ca^2+^ signals from all 113 neurons (left panel, grey line) arranged by the amplitude of the evoked LFPs (left panel, black line). The S.E.M. is shown. A significant correlation between the Ca^2+^ signals and the LFPs was observed (right panel, Pearson correlation, r = 0.77, p < 0.0001). (**c**) Plot of the transient counts for all 113 neurons (left panel, grey line) arranged by the amplitude of the evoked LFPs (left panel, black line). The S.E.M. is shown. A significant correlation between transient counts and LFPs was observed (right panel, Pearson correlation, r = 0.74, p < 0.0005).

To determine how well the Ca^2+^ responses were predicted by the LFPs, we compared the LFPs and the Ca^2+^ responses at both the individual neuron and ensemble levels. The whiskers were aligned in decreasing order (horizontal axis in Fig. 6b,c) based on the magnitude of the evoked LFP signals shown in Fig. 1c. The order of the whiskers was then changed into a red-blue colour scale: red corresponded to the C1 whisker, which evoked the largest LFP; blue corresponded to the E3 whisker, which evoked the weakest LFP; and the various shades of purple corresponded to the whiskers that evoked LPFs of intermediate magnitude. The normative topographical barrel map^41^ was then filled in using this colour scheme (left panel in Fig. 6a). The resulting map graphically depicts the broad whisker selectivity of the LFP signals, which is most likely due to the summation of electrical activity over wide cortical areas.

The whisker selectivity of individual neurons was well matched by the whisker selectivity of the LFPs. The whisker preference of all 113 individual neurons was indicated using the red-blue colour scale shown on the left normative barrel map of Fig. 6a. The resulting two-photon map of whisker preference is shown in the right panel of Fig. 6a. The neurons coloured in red preferred the same whisker that the LFP predicted as PPW (C1). The neurons coloured in blue preferred the non-PPW that evoked the weakest LFP (E3). The neurons coloured in various shades of purple preferred non-PPWs that evoked LFPs of intermediate magnitudes.

At the population level, the ensemble Ca^2+^ responses of the 113 neurons were compared to the LFP responses. The magnitudes of the Ca^2+^ responses (Fig. 6b) and the transient counts (Fig. 6c) were plotted against the whiskers, which were arranged according to the magnitude of the evoked LFPs (Fig. 6b,c, left panel). Most whiskers evoked comparable Ca^2+^ responses and LFP signals; however, a few exceptional whiskers, such as C3, δ, and A2, evoked Ca^2+^ responses that were larger than expected based on the LFPs (see Discussion). Both the Ca^2+^ signals (Fig. 6b, right panel, r = 0.77, p < 0.0001, Pearson correlation test) and the transient counts (Fig. 6c, right panel, r = 0.74, p < 0.0005, Pearson correlation test) were significantly correlated with the LFPs.

In summary, the evoked Ca^2+^ responses, measured either as the magnitude of the Ca^2+^ signals or as the number of transients, were highly correlated with the evoked LFPs at both the individual and population levels.

### Summary of data pooled from four animals

**Figure 7:**
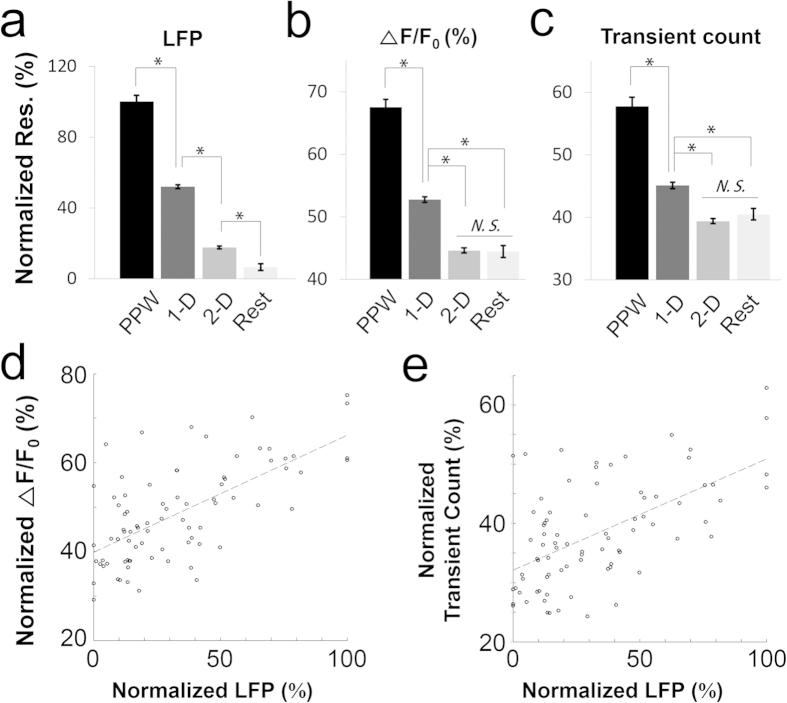
Summary. The data pooled from four animals are shown. The stimulated whiskers were classified into four groups: PPW, 1-D, 2-D, and Rest. 1-D indicates the whiskers surrounding the PPW. 2-D indicates the whiskers surrounding the 1-D whiskers. ‘Rest’ indicates all of the whiskers excluding the PPW, 1-D and 2-D groups. *p < 0.001. (**a**) Normalized LFP response to the stimulation of the PPW (total of 240 responses in 4 mice), 1-D (total of 1740 responses, 4 mice), 2-D (total of 2400 responses, 4 mice), and Rest (total of 420 responses, 4 mice) (Kruskal-Wallis one-way ANOVA on ranks, H = 1005.861, p < 0.001; Dunn’s method for multiple comparisons; PPW vs. 1-D: p < 0.001; 1-D vs. 2-D: p < 0.001; and 2-D vs. Rest: p < 0.001). (**b,c**) Normalized Ca^2+^ signals and calcium transients to stimulation of the PPW (total of 483 responses, 4 mice), 1-D (total of 3489 responses, 4 mice), 2-D (total of 4764 responses, 4 mice), and Rest (total of 841 responses, 4 mice) (Fig. 7b; Kruskal-Wallis one-way ANOVA on ranks, H = 418.122, p < 0.001; Dunn’s method for multiple comparisons; PPW vs. 1-D: p < 0.001; 1-D vs. 2-D: p < 0.001; and 2-D vs. Rest: p = 0.426) (Fig. 7c; Kruskal-Wallis one-way ANOVA on ranks, H = 190.342, p < 0.001; Dunn’s method for multiple comparisons; PPW vs. 1-D: p < 0.001; 1-D vs. 2-D: p < 0.001; and 2-D vs. Rest: p = 0.150) (**d**) The correlation between the LFP signals and the Ca^2+^ signals (total of 80 whiskers inspected from 4 mice, Pearson correlation, r = 0.66, p < 0.0001). (**e**) The correlation between the LFP signals and transient counts (total of 80 whiskers, Pearson correlation, r = 0.57, p < 0.0001).

A summary of the data pooled from four mice is shown in Fig. 7. All of the stimulated whiskers were classified into 4 groups: PPW, 1-D, 2-D and Rest. The whiskers that directly surrounded the PPW belonged to the ‘1-D’ group, and the whiskers that surrounded 1-D belonged to the ‘2-D’ group. The rest of the whiskers belonged to the ‘Rest’ group.

PPW stimulation evoked significantly larger LFP signals than stimulation of 1-D, 2-D and Rest whiskers (Fig. 7a; Kruskal-Wallis one-way ANOVA on ranks, H = 1005.861, p < 0.001; Dunn’s method for multiple comparisons; PPW vs. 1-D: p < 0.001; 1-D vs. 2-D: p < 0.001; and 2-D vs. Rest: p < 0.001; 240, 1740, 2400, and 420 individual LFP measurements for PPW, 1-D, 2-D, and Rest, respectively). All possible pairs among the 4 groups showed significant differences in LFP magnitude.

The PPW also evoked significantly stronger Ca^2+^ response than any other whiskers, as measured by the Ca^2+^ signals (Fig. 7b; Kruskal-Wallis one-way ANOVA on ranks, H = 418.122, p < 0.001; Dunn’s method for multiple comparisons; PPW vs. 1-D: p < 0.001; 1-D vs. 2-D: p < 0.001; and 2-D vs. Rest: p = 0.426; 483, 3489, 4764, and 841 neurons for PPW, 1-D, 2-D, and Rest, respectively) or the transient counts (Fig. 7c; Kruskal-Wallis one-way ANOVA on ranks, H = 190.342, p < 0.001; Dunn’s method for multiple comparisons; PPW vs. 1-D: p < 0.001; 1-D vs. 2-D: p < 0.001; and 2-D vs. Rest: p = 0.150; 483, 3489, 4764, and 841 neurons for PPW, 1-D, 2-D, and Rest, respectively). All possible pairs among the 4 groups showed significant differences in both Ca^2+^ response measurements, except for the pair of 2-D and Rest.

The Ca^2+^ responses showed a strong correlation with the LFP signals evoked by whisker stimulation (4, 29, 40, and 7 whiskers for PPW, 1-D, 2-D, and Rest, respectively). The correlation between the LFP signals and the Ca^2+^ signals was significant (Fig. 7d; Pearson Correlation, r = 0.66, p < 0.00001), as was the correlation between the LFP signals and the transient counts (Fig. 7e; Pearson Correlation, r = 0.57, p < 0.00001).

The LFP signals were significantly correlated with the Ca^2+^ responses evoked by whisker deflection. The LFP, therefore, could be used to predict the principal whisker of the area from which the LFP signal was obtained.

## Discussion

The LFP-based alternative provides a means as good as, and in some aspects better than, intrinsic-signal optical imaging. The biggest strength of the LFP-based method is the spatial precision of its targeting. Once the target is localized, no spatial adjustment in the position of the glass pipette is required before the calcium dye is ejected. As a result, the calcium dye can be loaded into the exact cortical area from which the LFP was obtained.

In contrast, targeting by intrinsic-signal optical imaging can produce significant positional errors. The low spatial resolution, combined with optical blurring induced by the imaging system, has the potential to cause a systematic positional offset^21^. In a simulation study using the standard parameters of intrinsic-signal optical imaging^21^, the systematic positional error of orientation pinwheels was estimated to be approximately 116 μm. Offline smoothing of the raw images by a spatial filter could add to this positional offset by an unpredictable amount (personal communication, K. Ohki). This error may explain why it has been difficult to match local functional structures such as ocular dominance columns, orientation pinwheels, and cytochrome oxidase blobs^42^,^43^.

Targeting based on intrinsic-signal optical imaging potentially has a secondary source of errors. After the target area is localized, the glass pipette containing the calcium dye needs to access the target area. Successful dye loading often requires visual monitoring of dye ejection by two-photon imaging. For this, an oblique penetration is required to avoid an objective above the pipette. Therefore, the insertion point of the pipette is displaced from the target area over the cortex. The amount of displacement needs to be carefully calculated, considering the angle of the pipette itself, the slope of the brain, and the desired depth for dye ejection. A significant miscalculation could result in the pipette missing the target. The LFP-based method requires none of these procedures, which results in more precise targeting than the optical method.

LFP-based targeting is also faster than targeting based on intrinsic-signal optical imaging. One of the biggest drawbacks of optical imaging based on intrinsic signal is its low signal-to-noise ratio. For example, an optimal visual stimulus could evoke a change in the signal of approximately 0.15%^44^. Brain movement, due to breathing and pulsation, can cause bigger signal changes. To overcome the problem of a low signal-to-noise ratio, raw images are collected through numerous repetitions and averaged afterwards. It may take tens of minutes to obtain only the raw images that are required for a typical orientation map of cat visual cortex. With the additional time spent changing the experimental setup (a CCD camera, a light source, and an objective), offline image processing to draw an orientation map, and pipette insertion after target localization, the intrinsic-signal optical imaging may take 0.5 ~ 1 hour before the dye is ejected. Any delay after opening the craniotomy window causes deterioration of the health of the brain and difficulty in loading the dye. In contrast, the LFP-based targeting does not delay the dye ejection; it does not require a change in the experimental setup, and it does not require the insertion of a new pipette towards the target. In addition, due to the relatively large LFP signals, less repetition is needed to identify the optimum stimulus.

Non-invasiveness might be the biggest strength of intrinsic-signal optical imaging. In contrast, LFP-based targeting can potentially damage the cortex if multiple trials are attempted. A researcher unfamiliar with barrel cortex may take many trials to find the target area. This not only delays the dye ejection but also causes damage to the cortex. However, there are two helpful factors. First, barrel topography is stereotypical. Only days are needed to become familiar with its topographical arrangement. Second, the oblique penetration increases the likelihood of obtaining the target barrel. With the angle we used (22 degrees), the pipette usually encountered 2-3 adjacent barrels in sequence during the penetration. This not only increases the chance of locating the target barrel during the first penetration but also helps to better orient the pipette over the barrel topography. This also greatly reduces the degrees of freedom in adjusting the spatial offset with respect to barrel topography for the next trial when the target was missed during the first penetration. In all four experiments, we were able to locate the target barrel in 1 or 2 trials.

The LFP-based method is limited in its application due to its low spatial resolution. Our results (left panel, Fig. 6a), as well as those of other studies^28^,^29^, indicate that LFP signals are summed over a volume of cortex that has a radius of a few hundred micrometres. Thus, the LFP-based method can only be used for cortical structures with spatial dimensions that match those of LFP signals, such as a specific location within a topographical map (e.g., a tonal band of the tonotopic map or a barrel of the barrel field) or a specific column of a functional map (e.g., an orientation column). LFP recording is unlikely to target very small cortical structures (e.g., anatomical microcolumns) or cortical areas that consist of neurons with heterogeneous response properties (e.g., an orientation pinwheel). However, if the impedance of the pipette is adjusted, the LFP recording could easily be applied to smaller structures. From LFPs to multiunits, electrophysiology-based targeting has the potential to localize functional structures which span a few millimetres to a few tens of micrometres.

The calcium responses we obtained after the LFP-based targeting were comparable to those obtained in previous studies using intrinsic-signal optical imaging. The amplitude of the averaged Ca^2+^ response to principal whisker stimulation (**ΔF/F** 1.155 ± 0.001%, mean ± S.E.M.) was comparable to the results of previous studies using intrinsic signal-based targeting (**ΔF/F** 1%)^19^. The small amplitude of the averaged Ca^2+^ response both in our data and in previous data might be due to the low response probability of layer 2/3 of the barrel cortex (0.11 APs/stim)^35^. The low response probability could be due to the anaesthesia used, as demonstrated by astrocyte calcium responses^45^. However, the response probability has been observed in the layer 2/3 barrel neurons of anaesthetized^20^,^35^,^46^ and awake, behaving rodents^47^,^48^ alike.

Our results revealed that, in both LFPs and Ca^2+^ responses, the layer 2/3 barrel neurons responded not only to the principal whisker but also to non-principal whiskers (Fig. 4a). This observation is consistent with previous studies; significant responses to whiskers other than the principal whisker have been observed for subthreshold membrane potentials^36^,^37^, single-unit action potentials^35^,^36^,^37^, and calcium signals in a two-photon imaging study^20^. Our results also showed that neurons located within tens of micrometres can have different preferred whiskers (Fig. 6a, right panel), and this result is also consistent with a previous calcium imaging study^33^. Previous extracellular recording studies have also reported that multiple neurons near the same electrode can exhibit different responses^49^,^50^. A previous study^35^ showed that a prominent response to the principal whisker was mostly observed in layer 4 rather than layer 2/3. Considering the inputs converged from layer 4 to layer 2/3, the response heterogeneity observed in our and previous studies is not surprising. This response heterogeneity of individual neurons in layer 2/3 might account for a few unexpected large calcium signals in ensemble response to the stimulation of non-PPWs such as C3, δ, and A2 (Fig. 6b, left panel).

We demonstrated that precise targeting of two-photon calcium imaging was successfully achieved using our proposed method. The LFP signal, obtained from the same glass pipette that was used for calcium dye ejection, was used to guide targeting. With this method, a specific barrel column of the mouse barrel cortex was precisely targeted, and the calcium dye was successfully loaded into the targeted barrel column, which led to successful *in vivo* two-photon calcium imaging of a cortical area with a volume of a few hundred micrometres.

## Methods

### Animal preparation and surgical procedures

1. All animal experiments were performed in accordance with a protocol approved by the Institutional Animal Care and Use Committee (IACUC) of the Korea Institute of Science and Technology. All methods were performed in accordance with relevant guidelines and regulations.
2. Four C57BL/6J mice (10 ~ 11 weeks of age) were anaesthetized with urethane (10% in physiological saline, 1.5 g/kg, IP). The depth of anaesthesia was monitored by the toe pinch at regular intervals. An additional dose of urethane was given if the animal showed a withdrawal response to the toe pinch. Once the animal was anaesthetized, it was placed in a custom-designed stereotaxic device. After the surgery, the anaesthesia was maintained by continuous automated injection of urethane (0.1 g/kg/h, IP, KDS100 from KD Scientific).
3. The skin above the skull was incised after the application of 2% lidocaine. The skull was exposed, cleaned, and dried. A custom-made metal plate, with a hole for the craniotomy, was then attached to the skull with dental cement (Grip Cement, Caulk/Dentsply International). A millimetre-high wall of dental cement was built around the craniotomy window to hold the ACSF during LFP recording or the distilled water during two-photon imaging, inside of the wall. The reference electrode for LFP recordings was attached to the skull near the craniotomy window.
4. The craniotomy was opened over the left barrel cortex (a 2 × 2 mm square centred at 2 mm posterior to Bregma and 3.5 mm lateral from the midline). The exposed cortex was superfused with ACSF containing (in mM) 135 NaCl, 5.4 KCl, 1 MgCl_2_, 1.8 CaCl_2_, and 5.0 HEPES, with its pH adjusted to 7.4 with KOH.
5. After the calcium dye was ejected, the craniotomy window was filled with agarose (1.5 ~ 2% in ACSF, Type III-A, Sigma-Aldrich) and sealed with a glass coverslip (World Precision Instruments). After allowing 0.5 ~ 1 hour for dye loading, two-photon imaging followed.
6. Rectal temperature was monitored and was maintained at 37.4 °C by a heating blanket (Harvard Apparatus) throughout the surgery and the experiment that followed. The quality of dye loading critically depends on the general health of the experimental animal.

### Calcium indicator

1. A glass pipette (1.2 mm OD with filament, World Precision Instruments) was pulled to a tip diameter of 4 ~ 5 μm by a pipette puller (P-97, Sutter Instruments). Oregon Green 488 BAPTA-1 AM (OGB-1 AM) was dissolved in DMSO with 20% pluronic acid. Sulphorhodamine (SR-101) was added to distinguish astrocytes from neurons^40^. SR-101 was useful for visualization of the dye ejection as well. This solution was then diluted in a pipette solution containing (in mM) 150 NaCl, 2.5 KCl and 10.0 HEPES, with its pH adjusted to 7.4 with KOH. The final concentrations were 0.8 mM for OGB-1 AM and 0.05 mM for SR-101. All chemicals were obtained from Molecular Probes or Sigma.
2. The final dye solution was sonicated for 10 minutes and filtered with a centrifuge filter (0.45 μm pore size, Millipore).

### Localization of the target area by LFP and dye ejection

1. The glass pipette was filled with the prepared dye and held by a pipette holder with two outlets: one for recording the LFP and the other for passing pressurized air (Figs 1a and 2a; 64-1237, Warner Instruments). The impedance of the pipette was 1.6 ~ 1.8 MΩ.
2. The electrical signal was band-pass filtered (1 ~ 300 Hz) and amplified (100×) (Model 1800, A-M Systems). An additional noise eliminator removed 50/60 Hz line noise (Hum Bug, Quest Scientific). The LFP signal was then digitized (PCI-6251, National Instruments). Custom-written LabView software was used for whisker stimulation in synchrony with LFP acquisition or two-photon imaging.
3. Based on the mouse atlas^51^, the glass pipette containing the calcium dye was inserted into the craniotomy window with a micromanipulator (MP-285, Sutter Instruments) at an angle of 22 degrees from the cortical surface. While advancing the pipette diagonally into the cortex, we used a visual display and an audio speaker to monitor LFPs evoked by whisker deflections that were manually applied at regular intervals. The optimal whisker was identified based on the large magnitude and short latency of the evoked LFPs. Whenever the optimal whisker seemed to match the target whisker, we focused on stimulating several whiskers with the target whisker in the centre. The pipette was positioned within the area where the optimal whisker matched the target whisker. This process took less than 5 minutes.
4. With the angle used, the tip of the pipette usually travelled 700 ~ 800 μm over the cortical surface until it reached the depth for dye ejection (250 ~ 300 μm). While the pipette tip travelled towards the proper depth, it usually encountered 2–3 adjacent barrel columns. If the target barrel was not encountered during the penetration, the pipette was withdrawn and reinserted into an adjusted location that was estimated by comparing the previously encountered barrel columns and the barrel topography^38^,^52^. With a little experience, it was possible to locate the target barrel column in one or two trials in approximately 10 minutes in all four of the experiments included in this study.
5. When the pipette was located in the presumed target barrel, the calcium dye was immediately ejected from the same pipette used for LFP recording. Dye ejection was visually monitored by two-photon imaging of SR-101 contained in the dye solution (Fig. 2a). A good loading was associated with slow and gradual spreading of SR-101 in a symmetric, circular pattern. We tried to obtain this pattern of spread by adjusting the pressure and duration of the applied air. For most experiments, we used an air pressure of 1 ~ 5 psi for 60 ~ 90 seconds to eject the dye. However, the pressure varied considerably because the pipette tip was often slightly clogged with tissue. Once the ejection parameters were determined, the ejection was repeated 3 ~ 5 times at 2–3 minute intervals (Picospritzer III, Parker instruments). The dye loading took approximately 0.5 ~ 1 hour. This protocol loaded the calcium dye into a cortical volume with a diameter of 300 ~ 400 μm.
6. To validate the proposed method, we obtained an LFP profile in response to stimulation of 18 ~ 21 whiskers (see Methods, Whisker stimulation) while the calcium dye was loaded. For all four of the experiments we performed, the whisker that was qualitatively determined to be the optimum whisker turned out to be the target whisker.

### Two-photon imaging

1. Two-photon imaging was performed on a Leica TCS SP5 microscope coupled with a Chameleon Ultra (Coherent Systems) mode-locked Ti-sapphire laser (810 nm for OGB-1AM and 880 nm for SR-101).
2. Emission was split and detected in two channels: one for green (500–550 nm) and the other for red (575 ~ 650 nm). Fluorescence images were acquired through a 20x water-immersion objective (Leica HCX APO L 20 × 1.0; 1.0 NA); 512 × 512 pixel images were acquired at 0.76 Hz (all imaging data except Fig. 2c), while 512 × 16 pixel images were acquired at 13.16 Hz (Fig. 2c). Each pixel was approximately 0.76 μm.
3. At the beginning of the acquisition of each image frame, a trigger signal was generated and sent to another computer via a parallel port to synchronize whisker stimulation with LFP data acquisition.
4. The evoked calcium signals were acquired from a depth of 180 ~ 240 μm below the pial surface, which corresponds to layer 2/3 of the mouse barrel cortex. All images were acquired at a single focal plane.

### Whisker stimulation

1. Whiskers were trimmed to 10 mm in length. Approximately 5 mm of the whisker was trapped in a glass tube attached to a piezo wafer (the black plate in Fig. 1A, PL128.11, Physik Instrumente).
2. The piezo wafer was controlled by a piezo amplifier (E-650 LVPZT, Physik Instrumente). A square-wave pulse was generated, smoothed by a software-based Bessel filter to avoid ringing, and fed into the piezo amplifier. The gain of the piezo amplifier was set to a value for which the piezo wafer deflected the end of the glass tube by approximately 650 μm. With this configuration, the whisker was deflected without interference from the neighbouring whiskers.
3. Whiskers were deflected at a low frequency (<1 Hz) to avoid fast sensory adaptation^53^. To validate the LFP-based targeting, 18 ~ 21 whiskers, including the target whisker, were stimulated in a random order. To obtain the LFP response profile, each whisker was deflected 30 times at 0.5 Hz (30 up and 30 down, 60 deflections over 1 minute; top trace in Fig. 1B). To obtain a calcium response profile by two-photon imaging, each whisker was deflected twice at 0.76 Hz during an on period that followed an off period (bottom blue trace in Fig. 2c). Each on or off period lasted for 5.256 seconds. Each whisker was deflected 20 times for 10 on periods (20 up and 20 down, 40 deflections over 105.12 seconds). It took 18 ~ 21 minutes to obtain the LFP response profile and 31.5 ~ 36.8 minutes to obtain the calcium response profile.

### Data analysis

1. A total of 4 mice were used in the analysis. For the LFP recording, 80 whiskers were stimulated in 4 mice. For calcium imaging, 480 neurons that were pooled from 4 mice were analysed. The data shown in Figs 1, 2, 3, 4, 5, 6 was obtained from a single mouse (Fig. 1 for LFP recording; Figs 2 ~ 4 for the calcium signals of individual neurons; Fig. 5 for the population calcium signals; Fig. 6 for the correlation of the population calcium signals with the LFPs). The summary shown in Fig. 7 is for the data pooled from all 4 mice.
2. In all 3D plots, the LFP amplitude is the averaged amplitude from 30 whisker stimulations.
3. Two-photon images were analysed with MATLAB scripts that were originally written by the Reid lab at Harvard Medical School^5^. The reference image was calculated by averaging all of the images obtained during the experiment. Occasional horizontal drifts of the images were corrected by shifting the image in the direction that maximized its correlation with the reference image. The contours of dye-loaded cells were detected automatically from the reference image and were manually corrected if necessary. Astrocytes were also automatically identified from the SR-101 image and excluded from further analysis.
4. The fluorescence intensity, F, was calculated by summing the pixel values within the contour for each neuron. The Ca^2+^ signal was defined as ΔF/F_0_, in which ΔF = F − F_0_. The baseline intensity, F_0_, was obtained by averaging the intensity from all of the off periods. The slow drift in the baseline intensity (over minutes) was removed by a high-pass filter (3- to 4-minute cut-off).
5. To detect the events in Fig. 2c, the original calcium signal was filtered with a low-pass filter (Butterworth; cut-off frequency, 0.8 Hz; drawn with a red line). Then, the events were detected relative to the threshold, which was calculated as 1.5 times the standard deviation of the raw signals from all of the off periods.
6. The Ca^2+^ signals shown in Figs 3a and 4a were obtained during 10 off-on stimulation periods for each whisker. Each on period contained 2 whisker movements, as described previously. The Ca^2+^ signals were obtained separately for each whisker and then plotted together. For easy comparison, data points between the whiskers were connected.
7. The negative Ca^2+^ signal values were truncated to zero for clear interpretation of the 3D plots (Figs 3b, 4b, and 5c).
8. The transient counts in Figs 3c, 4c, and 5d were obtained by counting supra-threshold Ca^2+^ transients during on periods. The threshold was defined as 1.5 times of the standard deviation obtained from all of the off periods.
9. To merge the data from 4 mice in Fig. 7, the normalized response was calculated by dividing the difference between the raw value and the minimum value by the difference between the maximum value and the minimum value. For the LFPs, the maximum or minimum value was determined in each mouse from the average magnitude of the LFPs evoked by whisker stimulation. For the Ca^2+^ responses, the maximum or minimum value was determined in each neuron from the average of the Ca^2+^ responses evoked by whisker stimulation.
10. For statistical analysis, Student’s t-test and one-way ANOVA with Holm-Sidak post hoc multiple comparisons tests were used if the data passed a normality test and an equal variance test. If not, Mann-Whitney rank sum test and Kruskal-Wallis one-way ANOVA on ranks with Dunn’s post hoc multiple comparisons tests were applied instead.

## Additional Information

**How to cite this article**: Lee, J.-H. *et al.* LFP-guided targeting of a cortical barrel column for *in vivo* two-photon calcium imaging. *Sci. Rep.* **5**, 15905; doi: 10.1038/srep15905 (2015).

## Supplementary Material

Supplementary Information
