# Supplementary material for: LFP-guided targeting of a cortical barrel column for in vivo two-photon calcium imaging
Source: Sci Rep. 2015 Oct 29;5:15905. doi: 10.1038/srep15905 (PMC4625133; doi:10.1038/srep15905)
Supplement: Supplementary Information [file srep15905-s1.pdf]

LFP-guided targeting of a cortical barrel column for *in vivo* two-photon calcium imaging

Joon-Hyuk Lee[1,3], Hee-Sup Shin[1,3], Kwang-Hyung Lee[1*], and Sooyoung Chung[2*]

[1] Department of Bio and Brain Engineering, Korea Advanced Institute of Science and Technology (KAIST), Daejeon 305-701, Republic of Korea

[2] Center for Neuroscience, Korea Institute of Science and Technology (KIST), Seoul 136-791, Republic of Korea

[3] Center for Cognition and Sociality, Institute for Basic Science (IBS), Daejeon 305-811, Republic of Korea

Author email:

Sooyoung Chung sooyoung@kist.re.kr

Kwang-Hyung Lee khlee@kaist.ac.kr

Co-corresponding authors (indicated by asterisks): Sooyoung Chung and Kwang-Hyung Lee

Sooyoung Chung: sooyoung@kist.re.kr, (TEL) +82-2-958-6954 , (Fax) +82-2-958-6937

Address: KIST L7314, Hwarang-ro 14-gil 5, Seongbook-gu, Seoul 136-791, Republic of Korea

Kwang-Hyung Lee: khlee@kaist.ac.kr, (TEL) +82-42-350-4313, (Fax) +82-42-350-8680

Address: KAIST E16, 291 Daehak-ro, Yooseong-gu, Daejeon 305-701, Republic of Korea

The authors declare no competing financial interests.

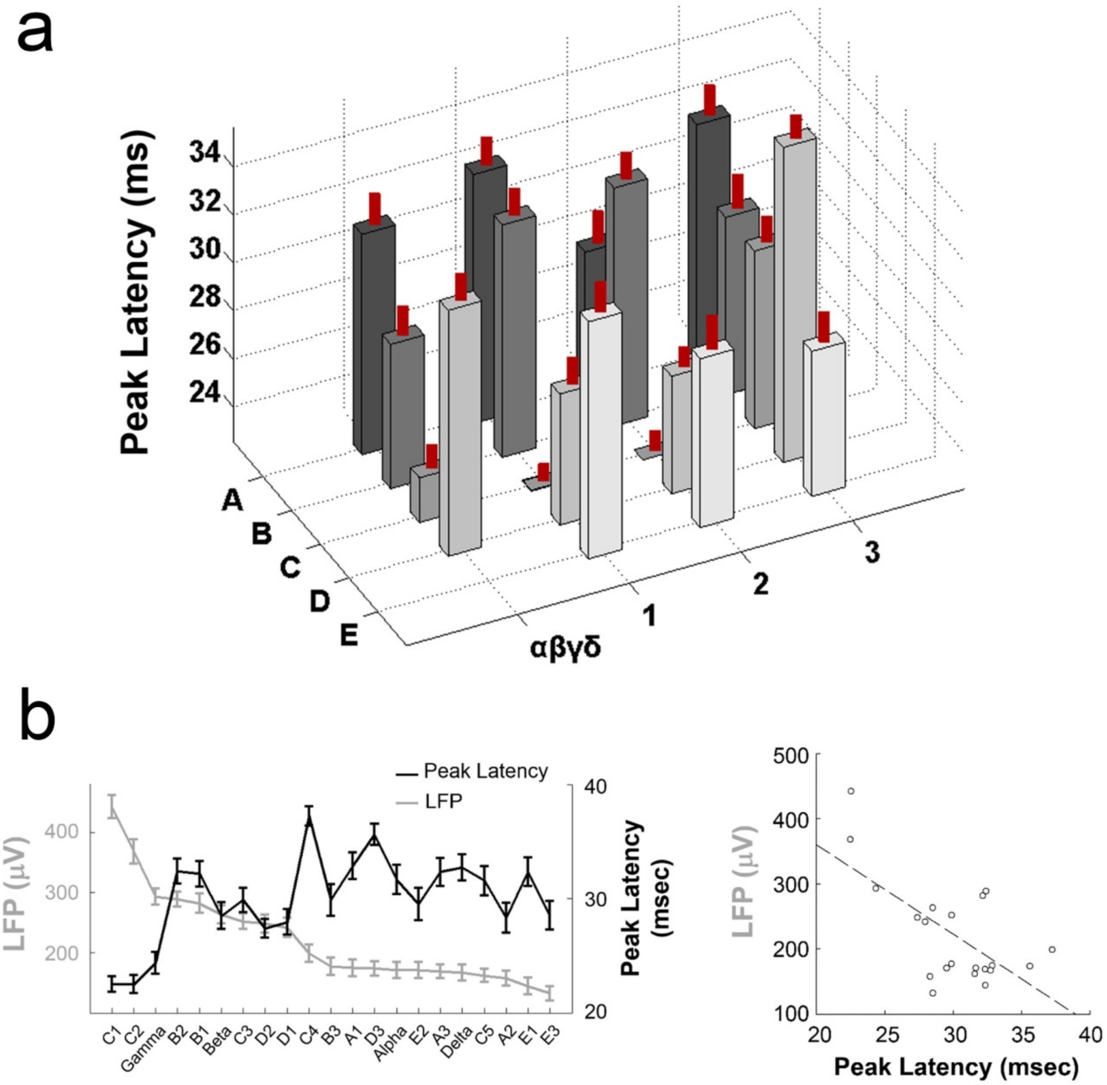

Supplementary Figure S1. Target barrel localization by LFP latency. (a) Topographical 3D plot of the latency of the peak of the LFP against the stimulated whisker. The latency of the peak response was obtained from the average LFP traces. Stimulation of the C1 whisker evoked a peak LFP with a significantly shorter latency than that evoked by stimulation of most of the other whiskers (Mann-Whitney rank sum test; A2: $p = 0.00021$, β: $p = 0.00007$, $p < 0.00001$ for whiskers α, A1, A3, B1, B2, B3, C3, δ, D1, D2, D3, E1, E2, and E3), excluding C2 and γ (C2: $p = 0.3655$; γ: $p = 0.073355$). (b) Relationship between LFP amplitude and LFP latency. A significant negative correlation was observed between the LFP amplitude and latency (right panel, Pearson correlation, $r = -0.656$, $p = 0.00123$).
